# Growth of Children with Type 1 Diabetes Mellitus

**DOI:** 10.4274/jcrpe.v2i2.72

**Published:** 2010-05-04

**Authors:** Korcan Demir, Ayça Altıncık, Ayhan Abacı, Atilla Büyükgebiz, Ece Böber

**Affiliations:** 1 Dokuz Eylül University, Department of Pediatric Endocrinology, İzmir, Turkey; 2 Keçiören Training and Research Hospital, Division of Pediatric Endocrinology, Ankara, Turkey; 3 Bilim University, Department of Pediatric Endocrinology, İstanbul, Turkey; +90 232 412 36 28+90 232 412 36 28ece.bober@deu.edu.trDepartment of Pediatric Endocrinology, Dokuz Eylul University, İzmir, Turkey

**Keywords:** growth, height, body mass index, type 1 diabetes mellitus, children

## Abstract

**Objective**: To retrospectively evaluate the effect of type 1 diabetes on growth.

**Methods**: Patients with Type 1 diabetes mellitus (T1DM) followed for at least one year after diagnosis, and without coexisting disorder that could affect growth, were included in this retrospective analysis. Height and body mass index (BMI) values were recorded. According to the data obtained at the end of each year of disease, the patients were divided into two groups: Group 1 (patients whose height standard deviation score (SDS) did not change or showed improvement) and Group 2 (patients whose height SDS showed a decline). The two groups were compared with respect to clinical and laboratory variables.

**Results**: Among the 248 patients followed, 101 (M/F:55/46) fulfilled the inclusion criteria. Overall, the mean height SDS of the patients did not change significantly during the follow−up period. BMI SDS showed no change during the course of the disease, except for a significant rise observed at the end of the first year compared to the value at diagnosis. Height SDS of the patients in Group 1 was higher compared to those in Group 2 from the 2^nd^ year of disease onwards (2^nd^ year, p=0.03; 3^rd^ year, p=0.02; 4^th^ year, p=0.01; 5^th^ year, p=0.03). The ratio of patients presenting with diabetic ketoacidosis at onset was significantly higher in Group 1 at the 4th year of diagnosis (p=0.03). Additionally, the mean HbA_1c_ level showed a modest negative correlation with Δ height SDS at the 3rd year of diagnosis (r=−0.3, p=0.03).

**Conclusions**: No significant deteriorative effect of T1DM on auxological parameters was observed at short term. Some clinical and laboratory variables related with metabolic control were found to correlate with growth.

**Conflict of interest:**None declared.

## INTRODUCTION

Type 1 diabetes mellitus (T1DM) is a chronic disorder with well−known short− and long−term consequences (1). One of the long−term consequences is severe impairment of growth and development, the so−called Mauriac syndrome (2). As a result of the major advances in diabetes care, this entity has now become a rarity. Indeed, during the last decade, there are some studies reporting positive growth characteristics in diabetic children (3, 4). However, growth deceleration during the course of the disease has been reported in various countries around the world, such as Austria, Brazil, Czech Republic, Germany, and Sudan (5, 6, 7, 8). The extent of the impact of clinical and laboratory variables on the growth of children with T1DM is also a controversial issue.

In this study, we aimed to evaluate the effect of T1DM on growth and body mass index (BMI) in children followed in the Department of Pediatric Endocrinology at Dokuz Eylul University.

## METHODS

**Setting and Subjects**

This study was conducted in the Department of Pediatric Endocrinology, Dokuz Eylul University, Izmir, Turkey. The hospital is one of the two tertiary referral university centers in the Aegean region of Turkey. The records of children with T1DM were evaluated retrospectively. A total of 248 patients with T1DM have been followed since the establishment of our department in 1990. The subjects that fulfilled the following inclusion criteria were enrolled in the study: (1) being diagnosed in our center, (2) a follow−up duration of at least 1 year, (3) not having achieved near−final height at presentation and (4) absence of coexisting disorder, such as celiac disease, autoimmune thyroiditis, that could affect growth. The study consisted of 101 patients (55 males, 46 females; 17 diagnosed between 1992 and 2000, 84 diagnosed between 2000 and 2007).

**Clinical and Laboratory Evaluation**

In general, diabetic children are followed at 3−month intervals in our Department. At each clinic visit, the following assessments are performed: complete physical examination, evaluation of capillary blood glucose measurements done at home, blood and urine sampling, and improvement in diabetes knowledge by a team consisting of a pediatric endocrinologist, a diabetes nurse, and a diabetes dietitian.

A Harpenden fixed stadiometer was used to measure the standing height (cm) to the nearest 0.1 cm. Body weight (kg) measurements were performed with a SECA scale sensitive to 0.1 kg, with subjects dressed in a light T−shirt and shorts. BMI was calculated as weight (kg) divided by the square of height (m). Auxological data were converted to standard deviation scores (SDS) using data from the National Health and Nutrition Examination Survey (9). The heights of the parents were either measured or recorded via telephone contact. The target height was computed as mean parental height in centimeters, +6.5 cm for boys and −6.5 cm for girls.

In our clinic, the tests for HbA1c and urine analyses are obtained every three months. Unless clinically indicated, routine analyses for complete blood count, liver and kidney function tests, lipid profiles, thyroid function tests, and celiac antibodies are made annually. Presence of microvascular complications (24−hour microalbumin excretion, eye fundoscopy, and electromyography) is also assessed regularly. HbA1c values were determined by Abbott Architect ci8200 system. HbA1c values between 4.5−6% were defined as normal.

**Analysis**

The patients meeting the inclusion criteria were evaluated with regard to mean height and BMI SDS at the end of each year of disease only during the first five years of follow−up, since the number of attending patients decreased in subsequent years. Thus, according to the data obtained at the end of each year of disease in the first five years of follow−up, the patients were grouped as: those whose height SDS did not change or improved since diagnosis (Group 1) and those who showed a deterioration in their height SDS since diagnosis (Group 2). These groups were compared regarding gender, age at diagnosis, height SDS, Δ height SDS (current height SDS – height SDS at presentation), target height SDS, BMI SDS, Δ BMI SDS (current BMI SDS−BMI SDS at presentation), pubertal state, clinical picture at presentation, and also regarding dominant mode of therapy, mean daily insulin dose, mean basal/total daily insulin ratio, and mean HbA1c level since the onset of the disease. Correlations of Δ height SDS at the end of each year of disease with Δ BMI SDS and long−term values of insulin dose, basal/total daily insulin ratio, and HbA1c levels were also assessed.

**Statistical Evaluation**

The data were statistically analyzed using computer software SPSS 11.0 (Chicago, IL, USA). All analyses were performed after ascertaining that the data were normally distributed using the one−sample Kolmogorov−Smirnov test. The group means and standard deviations were determined using descriptive statistics. Paired−samples t−test and repeated measures ANOVA test were used to assess the course of auxological variables. Group 1 and 2 were compared regarding the influence of the above−mentioned clinical and laboratory variables using Student’s t and chi−square tests. Univariate correlation analysis included all patients and was performed using Pearson’s test to identify the degree of correlation of numerical variables with Δ height SDS. A p−value of <0.05 was chosen to represent statistical significance.

## RESULTS

The mean age at diagnosis was 8.9±3.5 years (range, 2−15.5 years) and the mean duration of follow−up was 4.1±2.5 years (range, 1−13 years). Mean and standard deviation values for height and BMI SDS at diagnosis and during the follow−up period are presented in Table 1. The mean values for auxological variables at the time of diagnosis of the 101 patients (M/F:55/46) were as follows: height SDS 0.3±1.1, BMI SDS −0.6±1.4. At the time of diagnosis, both sexes were found to be taller compared to their target height [males (n=46), target height SDS −0.16±0.8 vs. height SDS at diagnosis 0.35±1.1, p=0.001 and females (n=42), target height SDS −0.24±0.8 vs. height SDS at diagnosis 0.04±0.8, p=0.04].

Table 2 presents the results of comparisons performed for various follow−up durations. The mean height SDS of the patients did not change significantly in any of the follow−up durations. All comparisons between BMI SDS values at the start and at the end of separate follow−up periods showed significant differences. In fact, these differences resulted solely from regaining the weight, which was lost during the onset of disease, especially during the first year.

There was no significant change in the mean values of height SDS during the first 5 years of the disease; however, comparisons between Groups 1 and 2 revealed significant differences in height SDS values from the 2^nd^ year of disease onwards (p=0.03, p=0.02, p=0.01, p=0.03, respectively) (Table 3). Presenting with diabetic ketoacidosis (DKA) at the onset was significantly associated with maintaining or gaining height SDS by the 4^th^ year of diagnosis (p=0.03). On the other hand, the mean HbA1c level showed modest negative correlation with Δ height SDS at the 3rd year of follow−up (r=−0.3, p=0.03). Remaining comparisons between the groups (mean target height SDS, rate of pubertal patients, mean daily insulin dose, mean basal/total insulin ratio, and mean HbA1c) or correlation analyses including the whole study group revealed no other significant association with Δ height SDS.

**Table 1:**
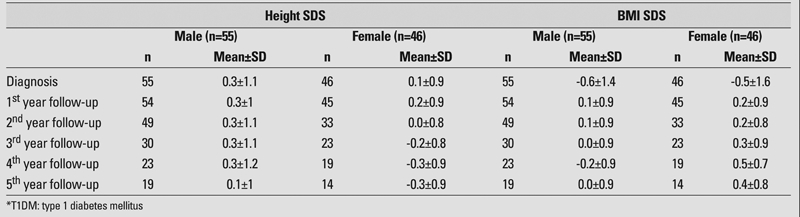
Mean values for auxological variables at diagnosis and during follow−up in T1DM* patients

**Table 2:**
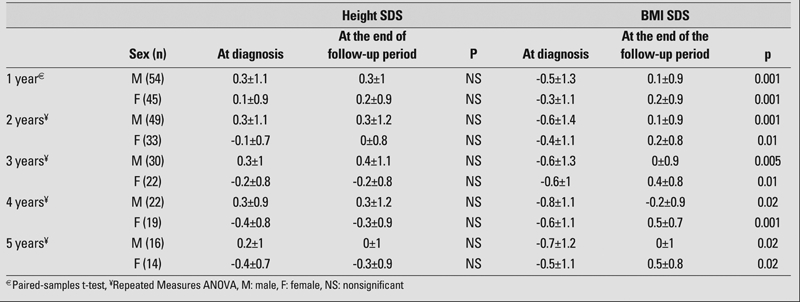
Results of the statistical analyses* in height and BMI SDS in T1DM patients at different follow−up years

**Table 3:**
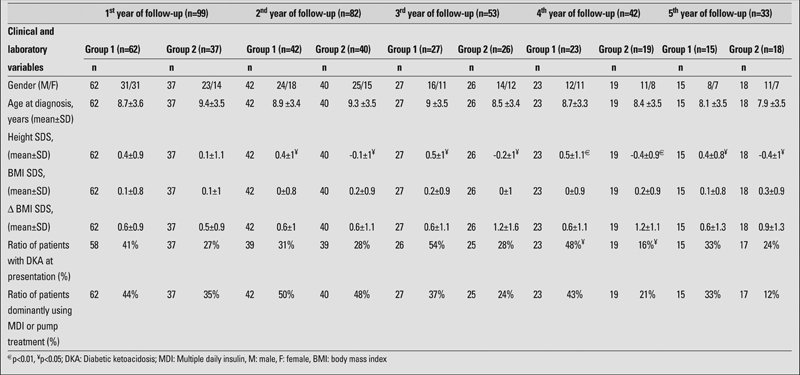
Comparisons between Group 1 (patients whose height values improved) and Group 2 (patients whose height values deteriorated) by the end of five separate follow−up periods

## DISCUSSION

At the time of diagnosis, our patients (both males and females) were significantly taller compared to their genetic target heights and slightly taller compared to reference values. It was first noted in the 1920’s that diabetic children have a tendency to be “over−height” on admission compared to normal children (10). In later years, conflicting results have been reported in over 30 studies on growth of children with T1DM. In 2002, DiLiberti et al (11) assessed the relevant data in a meta−analysis and concluded that the diabetic children were taller at the time of diagnosis attributing this finding to the taller parental stature. Our results, similar to the results of other studies, revealed that diabetic children were taller when compared to their midparental height SDS (8, 12, 13). Secular trend toward increased height might account for this difference (14). Recently, Larsson et al (15) demonstrated that a high human leukocyte antigen (HLA) risk for diabetes was correlated to an increased birth length SDS in children who later developed diabetes. However, this finding could not explain the increased postnatal height development of these children.

Studies evaluating the course of auxological variables following the diagnosis of T1DM generally report deterioration in height SDS. These early studies were conducted on children who were receiving conventional insulin treatment. However, Poyrazoglu et al (17) reported that final heights of the diabetic children followed between 1970 and 1987 were consistent with their target heights and no significant overall height loss was observed. Since early 1990’s, our understanding and the quality of management of T1DM have enormously improved (4, 5, 7, 8, 13, 16). In this study, we were able to evaluate the course of height SDS only in the first 5 years of follow−up and found that no significant change in height SDS occurred in both sexes. Bognetti et al (16) found that height SDS significantly decreased even in the first 3 years of disease in children and adolescents diagnosed between 1989 and 1992. Similarly, Donaghue et al (4) found loss of height SDS by the 5th year of disease in T1DM patients diagnosed between 1974 and 1991. However, similar to our findings, the 5th year height SDS of their patients diagnosed between 1991 and 1995 were similar to height SDS values at the time of diagnosis.

While the mean height SDS did not change during the first 5 years in our patients, we observed that some diabetic children maintained or gained (Group 1), while some diabetics lost height SDS (Group 2). Analyses comparing these children revealed two significant associations. First, presenting with DKA, which might have caused the patient and his/her family to be more conscientious, was more common in Group 1 by the 4th year of disease. Second, long−term mean HbA1c level had modest negative correlation with Δ height SDS by the 3^rd^ year of disease. Similar intra−group comparison was made by Danne (18), but at the age of 18. They found that longer diabetes duration, higher long−term mean HbA1c, and higher BMI at the age of 18 were significantly associated with a change in height SDS more than−1, compared to those who gained height SDS since diagnosis. However, we did not find any significant factors, other than the above−mentioned variables. Our inability to demonstrate any other statistically significant associations might be due to the decreasing number of patients, limiting the power of the statistical analysis. For instance, from the 3^rd^ year of disease onwards, noticeably higher percentage of patients in Group 1 were using multiple daily insulin injection or pump therapy compared to Group 2. On the other hand, there may be other uncovered factors for deterioration of height SDS, such as the effect of variations in blood glucose, which was shown to cause oxidative stress and epigenetic deteriorative changes, without affecting HbA1c levels (19, 20, 21).

In conclusion, no significant deteriorative effect of T1DM on auxological parameters in short term was observed in our patients. However, some clinical and laboratory variables related with metabolic control were found to correlate with growth.
